# The transposable element-rich genome of the cereal pest *Sitophilus oryzae*

**DOI:** 10.1186/s12915-021-01158-2

**Published:** 2021-11-09

**Authors:** Nicolas Parisot, Carlos Vargas-Chávez, Clément Goubert, Patrice Baa-Puyoulet, Séverine Balmand, Louis Beranger, Caroline Blanc, Aymeric Bonnamour, Matthieu Boulesteix, Nelly Burlet, Federica Calevro, Patrick Callaerts, Théo Chancy, Hubert Charles, Stefano Colella, André Da Silva Barbosa, Elisa Dell’Aglio, Alex Di Genova, Gérard Febvay, Toni Gabaldón, Mariana Galvão Ferrarini, Alexandra Gerber, Benjamin Gillet, Robert Hubley, Sandrine Hughes, Emmanuelle Jacquin-Joly, Justin Maire, Marina Marcet-Houben, Florent Masson, Camille Meslin, Nicolas Montagné, Andrés Moya, Ana Tereza Ribeiro de Vasconcelos, Gautier Richard, Jeb Rosen, Marie-France Sagot, Arian F. A. Smit, Jessica M. Storer, Carole Vincent-Monegat, Agnès Vallier, Aurélien Vigneron, Anna Zaidman-Rémy, Waël Zamoum, Cristina Vieira, Rita Rebollo, Amparo Latorre, Abdelaziz Heddi

**Affiliations:** 1 Univ Lyon, INSA Lyon, INRAE, BF2I, UMR 203, 69621 Villeurbanne, France; 2 Institute for Integrative Systems Biology (I2SySBio), Universitat de València and Spanish Research Council (CSIC), València, Spain; 3 Present Address: Institute of Evolutionary Biology (IBE), CSIC-Universitat Pompeu Fabra, Barcelona, Spain; 4 Laboratoire de Biométrie et Biologie Evolutive, UMR5558, Université Lyon 1, Université Lyon, Villeurbanne, France; 5 Department of Molecular Biology and Genetics, Cornell University, 526 Campus Rd, Ithaca, New York, 14853 USA; 6 Present Address: Human Genetics, McGill University, Montreal, QC Canada; 7 Department of Human Genetics, Laboratory of Behavioral and Developmental Genetics, KU Leuven, University of Leuven, B-3000 Leuven, Belgium; 8 ERABLE European Team, INRIA, Rhône-Alpes, France; 9 Present Address: LSTM, Laboratoire des Symbioses Tropicales et Méditerranéennes, IRD, CIRAD, INRAE, SupAgro, Univ Montpellier, Montpellier, France; 10 INRAE, Sorbonne Université, CNRS, IRD, UPEC, Université de Paris, Institute of Ecology and Environmental Sciences of Paris, Versailles, France; 11 Instituto de Ciencias de la Ingeniería, Universidad de O’Higgins, Rancagua, Chile; 12 Life Sciences, Barcelona Supercomputing Centre (BSC-CNS), Barcelona, Spain; 13 Mechanisms of Disease, Institute for Research in Biomedicine (IRB), Barcelona, Spain; 14 Institut Catalan de Recerca i Estudis Avançats (ICREA), Barcelona, Spain; 15 Laboratório de Bioinformática, Laboratório Nacional de Computação Científica, Petrópolis, Brazil; 16 Institut de Génomique Fonctionnelle de Lyon (IGFL), Université de Lyon, Ecole Normale Supérieure de Lyon, CNRS UMR 5242, Lyon, France; 17 Institute for Systems Biology, Seattle, WA USA; 18 Present Address: School of BioSciences, The University of Melbourne, Parkville, VIC 3010 Australia; 19 Present Address: Global Health Institute, School of Life Sciences, Ecole Polytechnique Fédérale de Lausanne (EPFL), 1015 Lausanne, Switzerland; 20 Foundation for the Promotion of Sanitary and Biomedical Research of Valencian Community (FISABIO), València, Spain; 21 IGEPP, INRAE, Institut Agro, Université de Rennes, Domaine de la Motte, 35653 Le Rheu, France; 22 Present Address: Department of Evolutionary Ecology, Institute for Organismic and Molecular Evolution, Johannes Gutenberg University, 55128 Mainz, Germany

**Keywords:** Coleoptera, Weevil, *Sitophilus oryzae*, Genome, Transposable elements, Endosymbiosis, Immunity, Evolution

## Abstract

**Background:**

The rice weevil *Sitophilus oryzae* is one of the most important agricultural pests, causing extensive damage to cereal in fields and to stored grains. *S. oryzae* has an intracellular symbiotic relationship (endosymbiosis) with the Gram-negative bacterium *Sodalis pierantonius* and is a valuable model to decipher host-symbiont molecular interactions.

**Results:**

We sequenced the *Sitophilus oryzae* genome using a combination of short and long reads to produce the best assembly for a Curculionidae species to date. We show that *S. oryzae* has undergone successive bursts of transposable element (TE) amplification, representing 72% of the genome. In addition, we show that many TE families are transcriptionally active, and changes in their expression are associated with insect endosymbiotic state. *S. oryzae* has undergone a high gene expansion rate, when compared to other beetles. Reconstruction of host-symbiont metabolic networks revealed that, despite its recent association with cereal weevils (30 kyear), *S. pierantonius* relies on the host for several amino acids and nucleotides to survive and to produce vitamins and essential amino acids required for insect development and cuticle biosynthesis.

**Conclusions:**

Here we present the genome of an agricultural pest beetle, which may act as a foundation for pest control. In addition, *S. oryzae* may be a useful model for endosymbiosis, and studying TE evolution and regulation, along with the impact of TEs on eukaryotic genomes.

**Supplementary Information:**

The online version contains supplementary material available at 10.1186/s12915-021-01158-2.

## Background

Beetles account for approximately 25% of known animals, with an estimated number of 400,000 described species [1–3]. Among them, Curculionidae (true weevils) is the largest animal family described, comprising about 70,000 species [1, 4, 5]. Despite being often associated with ecological invasion and ecosystem degradation, only three Curculionidae genomes are publicly available to date [6–8]. Among the cereal weevils, the rice weevil *Sitophilus oryzae* is one of the most important pests of crops of high agronomic and economic importance (wheat, maize, rice, sorghum, and barley), causing extensive quantitative and qualitative losses in field, stored grains, and grain products throughout the world [9–11]. Moreover, this insect pest is of increasing concern due to its ability to rapidly evolve resistance to insecticides such as phosphine, a fumigant used to protect stored grains from insect pests [12–14].

**Fig. 1.**
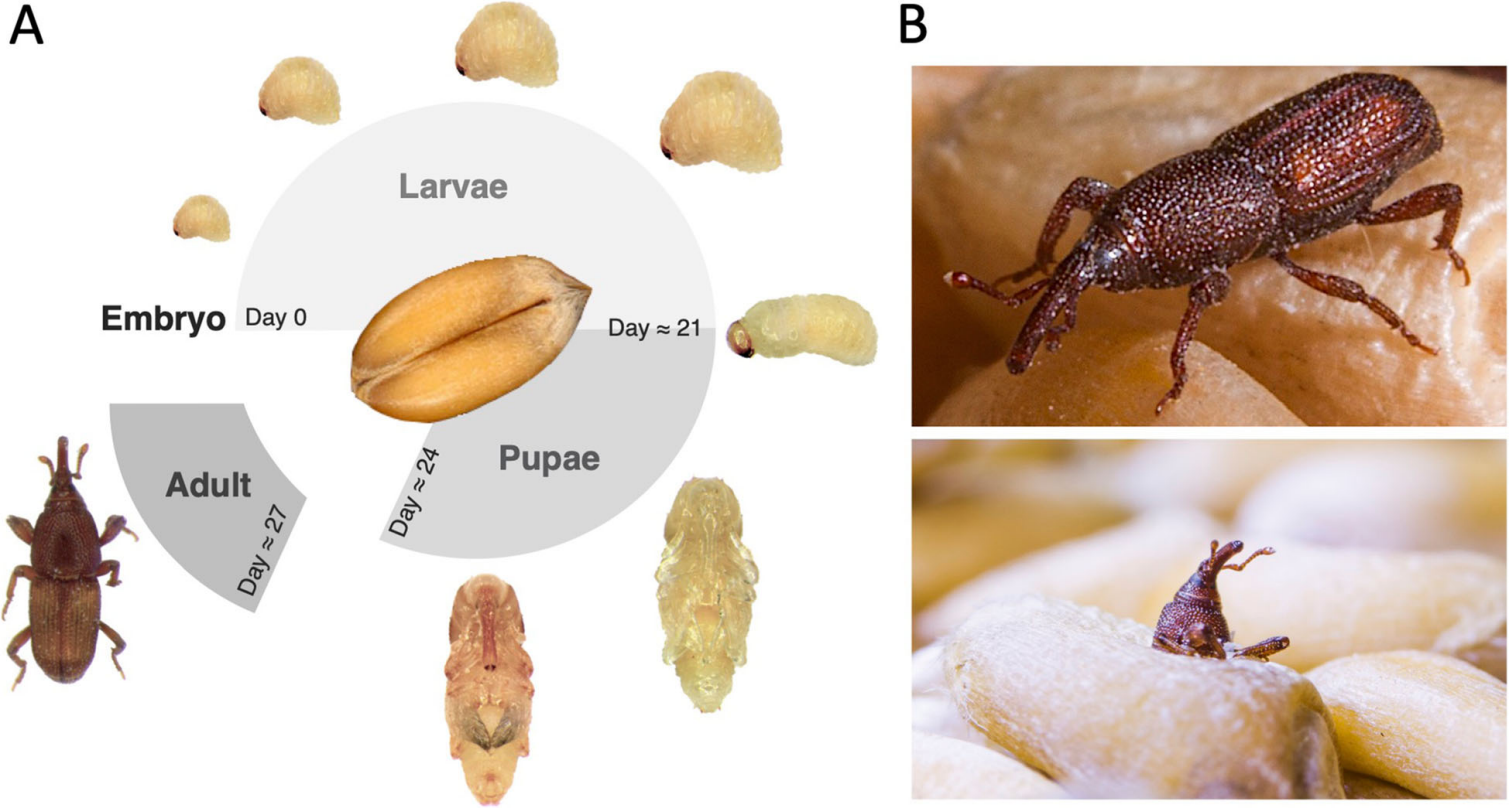
*Sitophilus oryzae* overview. **A** Life cycle of cereal weevil *Sitophilus oryzae*. The embryo develops into a larva and pupa, and metamorphoses into a young adult, exiting the grain around 3 days after metamorphosis completion. The developmental times indicated are from a rearing condition at 27 °C and 70% relative humidity. **B** Photos of adult *S. oryzae*. Lower panel shows an adult exiting the grain

Like other holometabolous insects, the life cycle of *S. oryzae* can be divided into four stages: egg, larva, pupa, and adult (Fig. 1). Females drill a small hole in the grain, deposit a single egg, and seal it with secretions from their ovipositor. Up to six eggs can be laid daily by each female, totaling around 400 eggs over its entire lifespan [15]. Larvae develop and pupate within the grain kernel, metamorphose, and exit the grain as adults. The whole process takes on average 30 days [10]. Like many insects living on nutritionally poor diets, cereal weevils permanently associate with nutritional intracellular bacteria (endosymbionts) that supply them with nutrients that are not readily available in the grains, thereby increasing their fitness and invasive power. The endosymbiont of *S. oryzae*, the gamma-proteobacterium *Sodalis pierantonius* [16, 17], is housed within specialized host cells, named bacteriocytes, that group together into an organ, the bacteriome [18]. Contrasting with most studied symbiotic insects, the association between *Sitophilus* spp*.* and *S. pierantonius* was established recently (less than 30,000 years ago), probably following the replacement of the ancestor endosymbiont, Candidatus *Nardonella*, in the Dryophthorinae subfamily [19, 20]. As a result, contrary to long-lasting endosymbiotic associations, the genome of *S. pierantonius* is GC rich (56.06%), and its size is similar to that of free-living bacteria (4.5 Mbp) [16]. Moreover, it encodes genes involved in bacterial infection, including type three secretion systems (TTSS), as well as genes encoding microbial associated molecular patterns (MAMPs) that trigger Pattern Recognition Receptors (PRR) and are usually absent or reduced in bacteria involved in long-lasting associations [16, 21, 22]. Nevertheless, many features indicate that the genome of *S. pierantonius* is in a process of degradation, as it contains many pseudogenes (43% of the predicted protein-coding sequences) and a large number of mobile elements (18% of the genome size) [16, 23]. Finally, it is important to note that no other symbionts, with the exception of the familiar *Wolbachia* endosymbiont in some strains, have been described in *S. oryzae*.

In order to help unravel potential adaptive functions and features that could become the basis for identifying novel control strategies for weevils and other major insect pests, we have undertaken the sequencing, assembly, and annotation of the genome of *S. oryzae*. Strikingly, the repeated fraction of *S. oryzae*’s genome (repeatome), composed mostly of transposable elements (TEs), is among the largest found to date in insects. TEs, the most versatile DNA units described to date, are sequences present in multiple copies and capable of relocating or replicating within a genome. While most observed TE insertions evolve neutrally or are slightly deleterious, there are a number of documented cases where TEs may facilitate host adaptation (for reviews, see [24–26]). For instance, gene families involved in xenobiotic detoxification are enriched in TEs in *Drosophila melanogaster* [27], *Plutella xylostella* [28], a major crop pest, and *Myzus persicae*, another phytophagous insect causing significant agronomic losses [29]. TEs have also been frequently associated with insecticide resistance in *Drosophila* species [30–32]. In addition, population genetics studies suggested that more than 84 TE copies in *D. melanogaster* may play a positive role in fitness-related traits [33], including xenobiotic resistance [32] and immune response to Gram-negative bacteria [34].

In eukaryotes, TE content varies drastically and contributes significantly to the size and organization of the genome. From TE-rich genomes as maize (85% [35]), humans (≈45% [36]), and the recently sequenced lungfish (≈90% [37]) for instance, to TE-poor genomes, as *D. melanogaster* (12–15% [38]), or *Arabidopsis thaliana* (≈10% [39]), repeatomes thrive on a high level of diversity. These drastic variations are also observed within animal clades, such as insects, where the proportion of TE ranges from 2% in the Antarctic midge (*Belgica antarctica*) to 65% in the migratory locust (*Locusta migratoria*) [40–42] and up to 75% in morabine grasshoppers (*Vandiemenella viatica* species) [43]. In addition to the overall TE content, the number of different TE families (homogeneous groups of phylogenetically related TE sequences), their size (number of copies per family), and sequence diversity are also very high among insect species [44]. For instance, SINEs (short interspersed elements) are almost absent from most insect genomes, but many lepidopterans harbor these elements [44]. In flies, long terminal repeat retrotransposons (LTRs) are a staple of the *Drosophila* genus, but such TEs are nearly absent from other dipteran genomes (e.g., *Glossina brevipalpis* and *Megaselia scalaris*) [44]. Recent advances in sequencing have dramatically increased the level to which TEs can be studied across species and reveal that such variations can persist even within recently diverged groups, as observed within *Drosophila* species [45], or among *Heliconius* butterflies [46]. An increasing number of insect genomes are reported with large repeatomes (e.g. *Aedes aegypti* and *Ae. albopictus* 40–50% [47, 48], *L. migratoria* 60–65% [40, 41], *Dendrolimus punctatus* 56% [49], *Vandiemenella viatica* species 66–75% [43]).

Here we present the genome of *S. oryzae*, with a strong focus on the repeatome, its largest genomic compartment, spanning over ≈74% of the assembly. *S. oryzae* represents a model system for stored grain pests, host-TE evolutionary biology, and the study of the molecular mechanisms acting at the early steps of symbiogenesis. Moreover, the features uncovered suggest that *S. oryzae* and its relatives have the potential to become a platform to study the interplay between TEs, host genomes, and endosymbionts.

## Results and discussion

### Genome assembly and annotation

We have sequenced and assembled the genome of the rice weevil *S. oryzae* at a base coverage depth of 142× using a combination of short- and long-read strategies (see “Methods” and Additional file 1). The assembly pipeline was defined to optimize multiple criteria including gene completeness (BUSCO scores [50]) and reference-free metrics (number of contigs, total length, N50, number of N’s per 100 kbp and the proportion of shared 100-mers between the assembly and short reads). The karyotype of *S. oryzae* comprises 22 chromosomes [51], and the genome assembly consists of 2025 scaffolds spanning 770 Mbp with a N50 of 2.86 Mbp, demonstrating a high contiguity compared to other Coleopteran genomes (Table 1). The assembly size is consistent with the genome size measured through flow cytometry (769 Mbp in females and 768 Mbp in males [51]). Haploid genome size estimations based on k-mer distributions of the short reads ranged from 785 Mbp (GenomeScope [58]) over 814 Mbp (gce [59]) to 818 Mbp (findGSE [60]), in agreement with the assembly size. BUSCO scores show the assembly is complete (97.9% complete and 0.7% fragmented), with a low duplication rate (1.9%). Consistent with the low duplication rate at the gene level, no significant haplotig contamination was observed. Finally, to confirm the completeness and consensus quality of *S. oryzae*’s assembly, we have firstly performed a K-mer analysis (100-mers), revealing that around 92% of the 100-mers of our assembly are covered by the 100-mers from the short reads, and secondly, 98% of the short reads were able to map to the assembly. Hence, thanks to the aforementioned statistics, *S. oryzae* is the best assembled Curculionidae genome to date [7, 52, 61] (Table 1). The complete analysis of gene content and function can be found in Additional files 2 and 3.

**Table 1:**
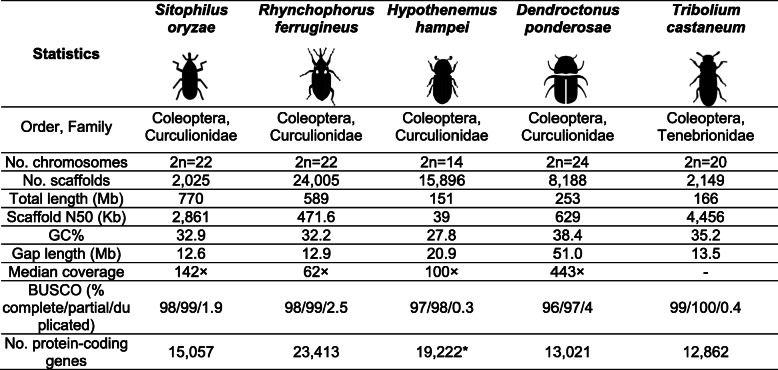
Assembly statistics of *S. oryzae*’s genome in comparison to Curculionidae genomes and *T. castaneum* [6, 7, 51–57]

*All genes, no NCBI RefSeq annotation report available

### Annotation of the *Sitophilus oryzae* genome

Among the different pathways we were able to decipher in the genome of *S. oryzae*, we present here highlights of the main annotation efforts, followed by a detailed analysis of the TE content and impact on the host genome. A comprehensive analysis for each highlight is presented as Supplemental Notes in Additional file 2.

#### Phylome and horizontal gene transfer

*Sitophilus oryzae* has a high gene expansion rate when compared to other beetles. Some of the families with the largest expansions include genes coding for proteins with DNA binding motifs, potentially regulating functions specific to this clade. Olfactory receptors, antimicrobial peptides (AMPs), and P450 cytochromes were expanded as well, probably in response to their ecological niche and lifestyle. Additionally, we noticed an expansion of plant cell wall-degrading enzymes that originated from horizontal gene transfer (HGT) events from both bacteria and fungi. Given the intimate relationship between *S. oryzae* and its endosymbiont, including the permanent infection of the female germline, we searched for evidence for HGT in the weevil genome possibly coming from *S. pierantonius*. Contrary to the genome of the tsetse fly *Glossina*, where at least three HGT events from *Wolbachia* have been reported [62], we were unable to pinpoint any HGT event from either the ancient endosymbiont *Nardonella*, *Wolbachia*, or the recently acquired *S. pierantonius*. A detailed description is reported in Additional file 2: Supplemental Note 1 [19, 63–86], and Note 3 for digestive enzymes [52, 87–107].

#### Global analysis of metabolic pathways

Using the CycADS [108] pipeline and Pathway Tools [109], we have generated BioCyc metabolism reconstruction databases for *S. oryzae* and its endosymbiont *S. pierantonius*. We compared *S. oryzae* metabolism to that of other arthropods available in the ArthropodaCyc [110] collection and we explored the metabolic exchanges between weevils and their endosymbionts. The metabolic reconstruction reveals that, despite its large genome for an endosymbiotic bacterium, *S. pierantonius* relies on its host for several central compounds, including alanine and proline, but also isocitrate, inosine monophosphate (IMP), and uridine monophosphate (UMP), to produce essential molecules to weevils, including the essential amino acids tryptophan, phenylalanine, lysine, and arginine, the vitamins pantothenate, riboflavin, and dihydropteroate as a folate precursor, and nicotinamide adenine dinucleotide (NAD) (Additional file 2: Supplemental Note 2). Among the amino acids listed above, phenylalanine, in particular, is an essential precursor for the cuticle synthesis in emerging adults [111]. In addition, several studies have shown that *S. pierantonius* improves host fitness, including fertility, developmental time, and flight capacity, in part by supplying the host with vitamins and improving its mitochondrial energy metabolism [112–114]. See Additional file 2: Supplemental Note 2 [19, 20, 108–110, 112, 113, 115–120] for more information.

#### Development

Developmental gene regulatory networks of *D. melanogaster* and *Tribolium castaneum* were used to annotate *S. oryzae* genes with roles in signaling, embryonic patterning, oogenesis, segmentation and segment identity, organogenesis, appendage and eye development, and insect size and developmental transitions. Overall, we observed a high level of conservation in comparison to the red flour beetle *Tribolium castaneum*, a model coleopteran. When compared to *D. melanogaster*, several key coordinate group genes are absent in *T. castaneum* and *S. oryzae*, most notably the anterior group genes *bicoid* and *swallow* and the posterior group gene *oskar*. Moreover, seven developmental genes with two homologs in the *Drosophila* genome are represented by a single ortholog in *T. castaneum* and *S. oryzae*. We also observed that homologs for signaling pathway ligands could not always be identified, which, given the presence of conserved receptors, is probably due to divergent primary sequence of the ligands. A detailed description is reported in Additional file 2: Supplemental Note 4 [52, 121–138].

#### Cuticle protein genes

Among the distinctive biological features of coleopterans is the ability to generate a hard and thick cuticle that protects them against dehydration and represents the first physical barrier from infections and topical insecticide penetration. The analysis of cuticle proteins (CPs) showed that *S. oryzae* has an average number of CPs, but with an enrichment of members of the CPAP1 family. While some members of this family are known to be involved in molting and maintaining the integrity of the cuticle in *T. castaneum*, most are still uncharacterized [139, 140]. Thus, these proteins might be involved in the development of specific cuticular tissues in *S. oryzae* or other weevils. The total number of CPs did not follow the taxonomy of beetles, suggesting instead that it might be an adaptation to their diverse lifestyles. For details, see Additional file 2: Supplemental Note 5 [139–143].

#### Innate immune system

The analysis of immunity-related genes revealed that the genome of *S. oryzae* encodes the canonical genes involved in the three main antimicrobial pathways Toll, Imd, and JAK-STAT, suggesting functional conservation of these pathways in cereal weevils. The conservation of the Imd pathway in the *S. oryzae* genome is of particular interest as its degradation in other symbiotic insects (*Acyrthosiphon pisum* [144], *B. tabaci* [145], or *Rhodnius prolixus* [146]) was initially correlated to their symbiotic status. The Imd pathway is not only present in *S. oryzae*, but it is also functional [147, 148], and has evolved molecular features necessary for endosymbiont control [147] and host immune homeostasis [148]. Thus, not only is the Imd pathway conserved in cereal weevils, contrary to aphids and some other hemimetabolous insects, but it seems to have been evolutionary “rewired” toward additional functions in symbiotic homeostasis [147]. A detailed description can be seen in Additional file 2: Supplemental Note 6 [41, 62, 144–193].

#### Detoxification and insecticide resistance

Fumigation using phosphine, hydrogen phosphide gas (PH_3_), is by far the most widely used treatment for the protection of stored grains against insect pests due to its ease of use, low cost, and universal acceptance as a residue-free treatment [194, 195]. However, high-level resistance to this fumigant has been reported in *S. oryzae* from different countries [13, 196–203]*.* Hence, we searched for genes associated with detoxification and resistance to insecticide and more generally to toxins, including plant allelochemicals. The *S. oryzae* repertoire of detoxification and insecticide resistance genes includes more than 300 candidates, similar to what is seen in other coleopteran genomes. For more details, see Additional file 2: Supplemental Note 7 [12–14, 110, 194–212].

#### Odorant receptors

One promising pest management strategy relies on modifying insect behavior through the use of volatile organic compounds that act on odorant receptors (ORs) [213, 214]. ORs play a significant role in many crucial behaviors in insects by mediating host-seeking behavior, mating, oviposition, and predator avoidance [215]. Interfering with the behavior of pest insects and modulating their ability to find suitable hosts and mates has been shown to reduce population numbers, notably using plants that are capable of producing attractants and repellents [216, 217]. *Sitophilus* spp. are known to use kairomones for host detection [218, 219], as well as aggregation pheromones [220, 221]. We annotated 100 candidate OR genes in *S. oryzae* (named SoryORs), including the gene encoding the co-receptor Orco. Of these genes, 46 were predicted to encode a full-length sequence. The global size of the SoryOR gene repertoire is in the range of what has been described in other species of the coleopteran suborder Polyphaga (between 46 in *Agrilus planipennis* and more than 300 in *T. castaneum*) and close to the number of OR genes annotated in the closely related species *Dendroctonus ponderosae* (85 genes, [222]) (Additional file 2: Supplemental Note 8 [204, 218–242]).

### Massive expansion of TE copies in the genome of *S. oryzae*

#### Detection and annotation of the repeatome

The repeatome represents the fraction of the genome categorized as repetitive. It encompasses TEs, satellites, tandem, and simple repeats. Eukaryotic TEs can be separated into two classes, depending on their replication mode [243]. DNA (Class II)-based elements are able to directly move within a genome and include terminal inverted repeat (TIR), Crypton, rolling circle (RC/Helitron), and large composite elements (Maverick). Conversely, retrotransposons (Class I) have an RNA intermediate and replicate through RNA retrotranscription. Retrotransposons can be further divided into long terminal repeat (LTR), and non-LTR elements, including long and short interspersed nuclear repeat elements (LINEs and SINEs). Other retrotransposons include Penelope-like (PLEs) and DIRS-like elements. Each one of these TE orders can be further classified into specific superfamilies (as for instance Copia or Gypsy LTR elements, and hAT or Tc1/Mariner TIR elements) that may encompass hundreds of TE families, each containing thousands of copies. The intrinsic diversity of TEs complicates their identification and annotation, especially in understudied species genera.

We used multiple state-of-the-art TE detection tools, including RepeatModeler2 and EDTA [244, 245], to generate consensus sequences of the TE families in *S. oryzae*. After an initial discovery step, more than 10,000 likely redundant TE families were identified by the dedicated programs; we combined their results using multiple sequence alignments and clustering (see “Methods” and Additional file 2: Figure S1) to reduce this number to 3399. After quality filtering (see “Methods”), the final library includes a total of 3361 sequences. Due to the evolutionary distance between *S. oryzae* and other known coleopterans, the consensus sequences obtained were further classified using a thorough combination of sequence homology and structure (see “Methods”). The *S. oryzae* genome is among the most TE-rich insect genomes to date. Comparison of TE genomic content as given by RepeatMasker using TE libraries from RepeatModeler 2.0.1, EDTA v1.7.8 or our custom pipeline shows that traditional methods miss ~ 5% of TEs in spite of harboring more complexity (more total TE consensus, Table S1). Thus, we conclude that our method is likely to improve the overall quality of the TE annotation.

**Fig. 2.**
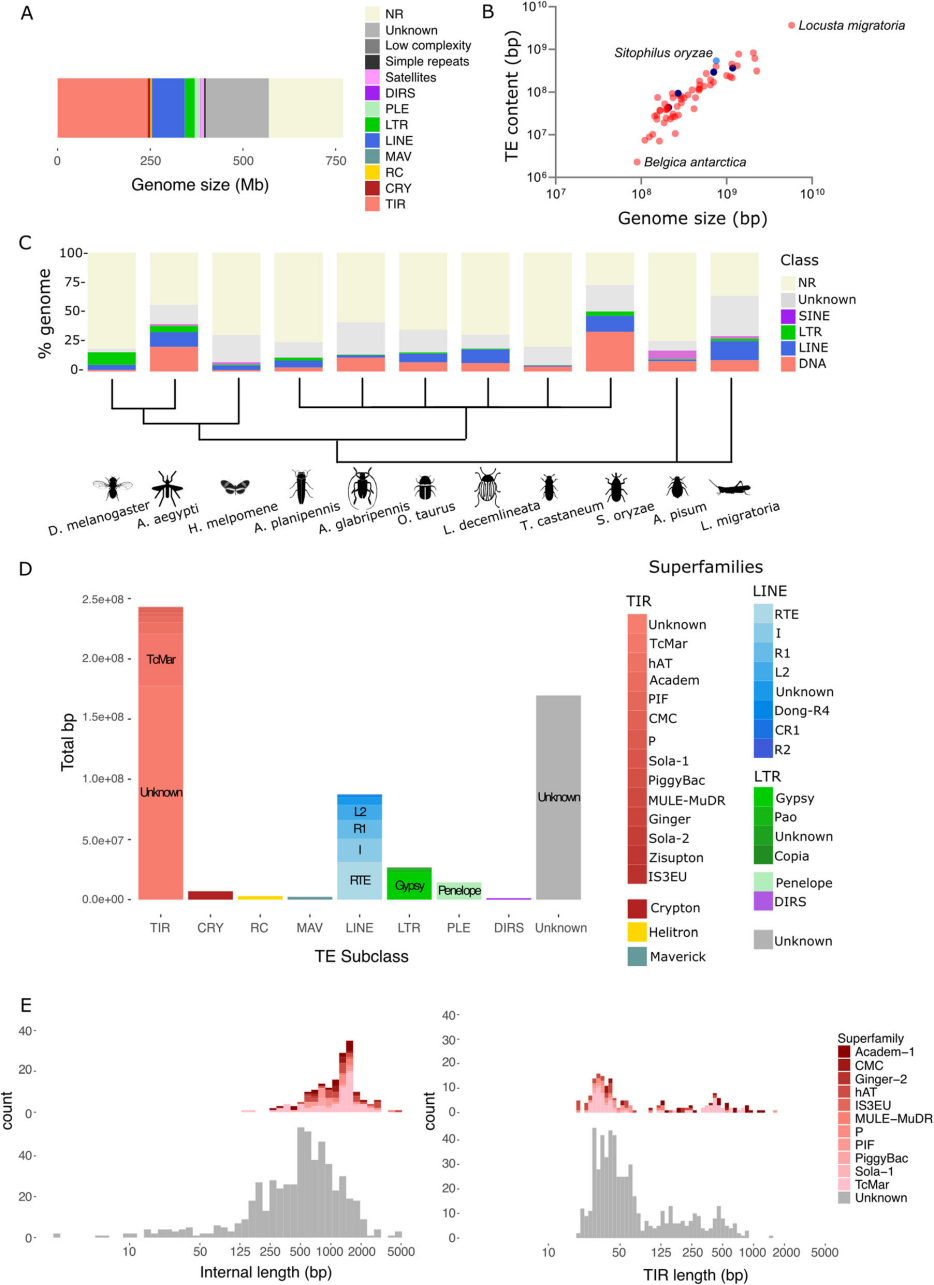
**A** Proportion of repeat content in *S. oryzae*’s genome. The majority of repeats detected in *S. oryzae* are represented by Class II (TIR) elements, LINEs (Class I), and unclassified repeats (unknown). NR: non repetitive. **B** Variation of genome size and TE content in 62 insect species from [40] and *S. oryzae*. Coleopteran species are depicted in dark blue, and *S. oryzae* in light blue. *S. oryzae* is clearly a TE-rich genome. **C** TE proportion across 11 insect species, including six coleoptera. In agreement with the data used for comparison [40], PLEs are included in the LINE superfamilies, DIRS in LTRs, and RC, CRY, MAV and TIR in the DNA superfamilies. NR: non repetitive. *S. oryzae* harbors the largest TE content among Coleopterans and most insect species studied to date. Within Coleoptera, there is a large variation in TE content and type, with *A. planipennis*, *L. decemlineata*, and *O. taurus* carrying an abundant LINE content, while *S. oryzae*, *T. castaneum*, and *A. glabripennis* show larger DNA content. Cladogram based on [246]. **D** Classification of the 570 Mbs of TEs present in the *S. oryzae* genome. Most TIR families detected were not classified into known superfamilies. RTE LINE and Gypsy LTR elements are the most abundant superfamilies among retrotransposons. Around 21% of repeats in *S. oryzae*’s genome were not classified by our pipeline, and remain unknown (gray). **E** Distribution of TIR length sequences (right) detected by einverted and the internal region present between both TIRs (left) for complete consensus of TIR superfamilies (color) and unknown TIR families (gray)

We uncovered 570 Mbp of repeat sequences, corresponding to ≈74% of the *S. oryzae* genome: ≈2% of satellite sequences, simple or low-complexity repeats, and ≈72% of other mobile elements, including TEs (Fig. 2A, Additional file 4). Given the limitation of the sequencing technologies, the proportion of satellites and TEs usually abundant in the heterochromatin is likely underestimated. We took advantage of a recent comparative analysis of TE content in 62 insect species [40] to contrast with the *S. oryzae* TE compartment. The *S. oryzae* genome ranks among those with the highest TE fraction observed in insects (Fig. 2B, C). Within the largest insect order, Coleoptera, very little is known regarding TE distribution and evolution. *T. castaneum* harbors only 6% of TEs [52] and *Hypothenemus hampei* contains 8.2% of TEs [6, 247], while *Dichotomius schiffleri* harbors 21% [248]. The species closest to *S. oryzae*, *Rhynchophorus ferrugineus*, has a TE content of 45% [8]. Therefore, while TE content has been described to follow phylogenetic relationships in most insects [44, 45], there is a large variation among the few Coleoptera species with available genomes. It is important to note that the pipeline we used to detect and annotate TEs in *S. oryzae* differs from the method implemented by Petersen and colleagues [40], as we incorporated 31 manually curated TE references for *S. oryzae*, and specifically annotated DNA/TIR elements based on their sequence structure (see “Methods”), increasing the annotation sensitivity.

#### Class II (DNA) elements dominate *S. oryzae*’s genome

The most striking feature of the genome of *S. oryzae* is the high abundance of Class II (DNA) elements (≈32% of the genome, ≈43% of the TE content) (Fig. 2A), which is the highest observed among all 62 insect species included in this analysis [40–42]. The most DNA transposon-rich genomes include mosquito *Culex quinquefasciatus* and *Ae. aegypti*, harboring 25% and 20% of DNA transposon content in their genome, amounting to 54% and 36% of the total TE compartment, respectively [6]. The TE-rich grasshopper *L. migratoria* repeatome comprises only 14% of DNA transposons, while LINE retroelements (Class I) amount to 25%. Morabine grasshoppers, with up to 75% of TE content, show equivalent amounts of DNA, LINE, and Helitrons [43]. Finally, among Coleoptera, a large diversity of repeatomes is observed (Fig. 2C) with *A. planipennis*, *Leptinotarsa decemlineata*, and *Onthophagus taurus* carrying an abundant LINE content, while *S. oryzae*, *T. castaneum*, and *Anoplophora glabripennis* show larger DNA transposon content.

Among the Class II elements present in *S. oryzae*, the majority belongs to the TIR subclass but has not been assigned a known superfamily (Fig. 2D), while Tc Mariners make up ≈6% of DNA elements. Among the consensus sequences, we were able to assemble from 5′TIR to 3′TIR (highest confidence, see “Methods”), the length distribution shows a continuum starting at a couple of hundred bases to a maximum of ≈ 5 kbp (see Fig. 2E). We hypothesize that most of the smaller TIR families observed are miniature inverted repeat elements (MITEs). MITEs are non-autonomous elements, deriving from autonomous Class II/TIR copies, comprising two TIRs flanking a unique, non-coding, region (sometimes absent) of variable length. While the TE detection pipeline used was able to detect and annotate most Class II/TIR elements based on transposase homologies, we also specifically searched for non-autonomous TIR sequences, allowing the detection of putative MITEs that lack protein-coding regions (Additional file 2: Figure S1). Among all Class II/TIR superfamilies, TIR length varies between tens of base pairs to ≈1 kbp (Fig. 2E). We identified short elements, composed mostly of their TIR sequences (Fig. 2E), typical of MITEs. Interestingly, the unknown TIR families show an average size smaller than 1 kbp, while TIRs with an annotated superfamily, show larger sizes (Additional file 2: Figure S3), suggesting that most unknown families could be indeed non-autonomous MITEs. MITE size ranges were previously described from around 100 bp to copies reaching more than 1 kbp [249]. Finally, the distribution of the proportions of TIR relative to the consensus length appears superfamily-specific (Fig. 2E and Additional file 2: Figure S3), and unknown families recapitulate these patterns. In conclusion, while most unknown TIR families seem to be composed of MITEs, we cannot exclude that our homology database is limited, likely missing some unknown protein domains. The most abundant TE family detected in the *S. oryzae* genome is indeed a MITE element (TE2641_SO2_FAM0704), with 10,486 genomic hits (or the equivalent of ≈4117 copies based on the consensus size), corresponding to 1.3% of the genome. Large fractions of MITEs were also reported in Class II-rich genomes, such as the aforementioned mosquitoes [48, 250] and the invasive *Ae. albopictus* [47], but also in many plant species such as the rice *Oryza sativa* [251–253]*.* Among Class II elements, we have also detected Crypton (0.9% of the genome), RC/Helitrons (0.4% of the genome). and Mavericks (0.3% of the genome).

LINE elements are the second most abundant TE subclass, representing ≈11% of the *S. oryzae* genome, among which ≈35% are assigned to RTE elements and ≈22% to I elements (Fig. 2D). No SINE families have been detected. LTRs are rather scarce, representing only ≈3% of the genome (Fig. 2D), and the vast majority belong to the Gypsy superfamily (≈30%). Another retrotransposon order detected are Penelope (PLEs), reaching nearly 2% of *S. oryzae*’s genome, and DIRS (tyrosine recombinase retrotransposons, 0.14% of the genome).

Finally, around 22% of the genome is composed of repeats for which our pipeline could not assign a known TE class (Fig. 2D). CDD search on peptides greater than 100 aa extracted from “Unknown” consensus found a total of 74 distinct hits (*P* ≤ 0.01), for a total of 50 consensus. We identified 14 unknown consensus with hits against known TE domains or viral sequences. The other 36 sequences had significant hits against Eukaryotic or Prokaryotic domains, traditionally not associated with TEs. Therefore, potential non-TE sequences within the unknown fraction represent an estimated total of 0.35% of the genome and were removed from the TE library. These unknown families highlight the wealth and diversity of TEs among insects and Coleopteran genomes in particular.

#### TE copies make up most of non-coding sequences of *S. oryzae*’s genome

**Fig. 3.**
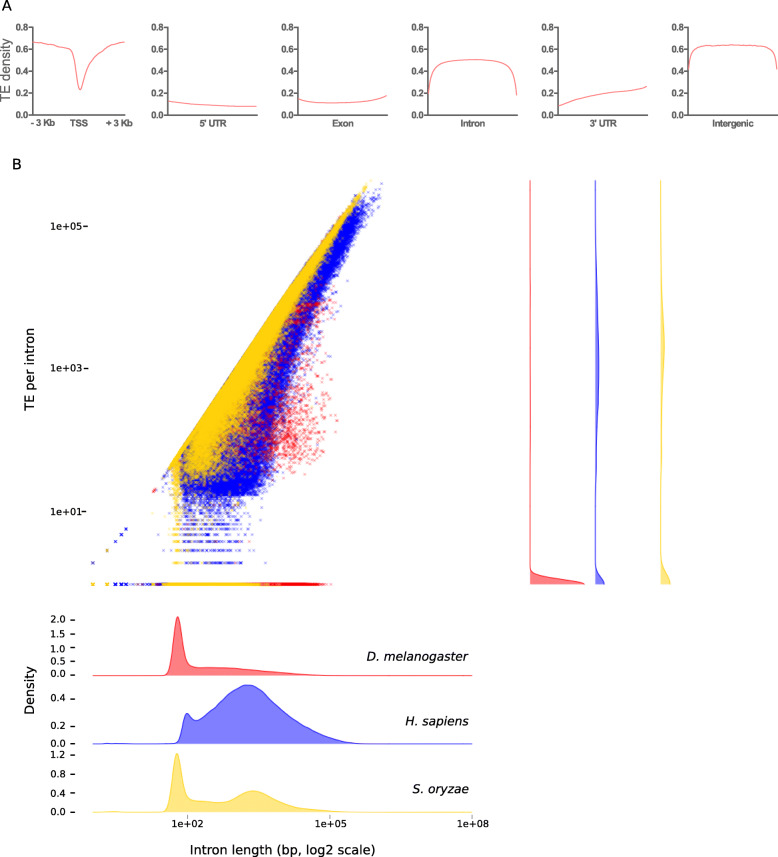
TE distribution in *S. oryzae*’s genome. **A** Density of TE copies within gene regions. TE copies are the least abundant within TSSs, 5′ and 3′ UTRs and exons, while introns and intergenic regions are riddled with TEs. TSS: transcription start site, UTR: untranslated regions. **B** Relationship between intron length and TE per intron in *D. melanogaster* (red), *H. sapiens* (blue), and *S. oryzae* (yellow). *S. oryzae* shares characteristics of both *Drosophila* with short and TE-poor introns and Humans with a significant number of large, TE-packed introns

TE copies are interspersed around the *S. oryzae* genome. TEs are less frequently found close to gene transcription start sites (TSS), 5′ and 3′ untranslated regions (5′ and 3′ UTRs) and exons (Fig. 3A), as expected. On the contrary, introns and intergenic sequences harbor the highest TE content (Fig. 3A), amounting to around 50% of TE density, close to the general TE proportion in the genome (72%), suggesting that most non-coding DNA sequences in the *S. oryzae* genome are virtually made of TEs. To grasp the impact of TEs on intron size, we compared intron length in *S. oryzae* with two very well assembled genomes: *D. melanogaster* with a very compact and small genome, and the large, TE-rich human genome (Fig. 3B). In *D. melanogaster*, introns are small and harbor few TEs, while in humans, introns are much larger potentially due to high TE accumulation [254]. *S. oryzae* intron sizes also seem to be due, at least partly, to TE accumulation. Interestingly, the *S. oryzae* genome presents a bimodal distribution, with a large proportion of small introns, as found in *D. melanogaster*, but also a noticeable amount of larger, TE-packed and more human-like introns. This could suggest that specific regions of the genome could be more prone to TE elimination, and be associated with high rates of recombination and/or signature of purifying selection.

#### TE activity inferred by evolutionary history

**Fig. 4.**
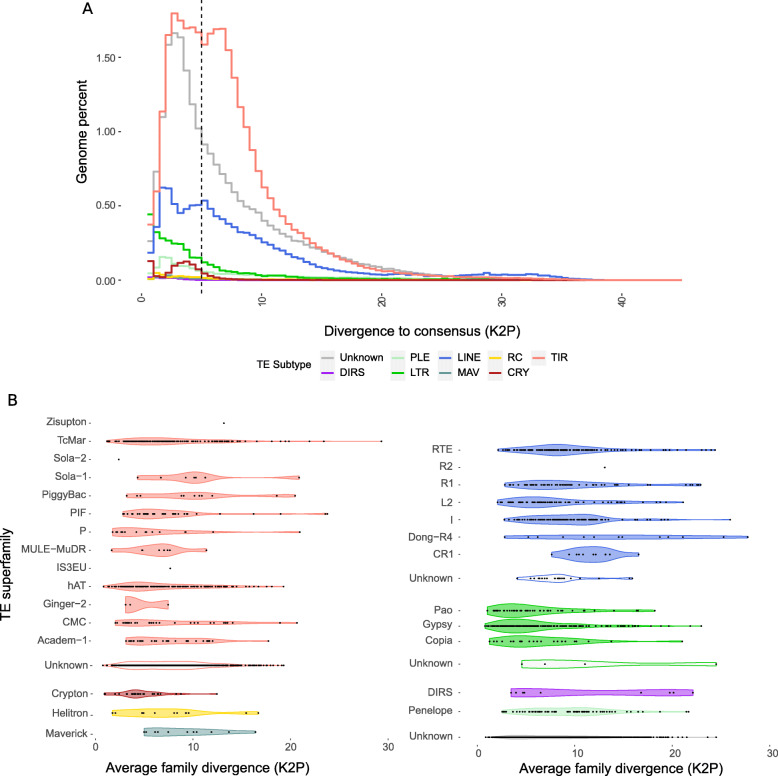
**A** TE divergence landscape. Distribution of the divergence (Kimura two parameters, K2P) between TE copies and their consensus, aggregated by TE class reported in percent of the genome. The less divergent superfamilies are distributed to the left and suggest recent activity. Strikingly, most of the TE copies have less than 10% divergence to their consensus, with a large number of copies under 5% (dotted line). The distribution of the “unknown” class overlaps with the leftmost mode of the TIR distribution, suggesting that many more TIR families are yet to be described in *S. oryzae.* Strikingly, LTR elements are the least diverged altogether with the mode of the distribution on the 0–1% divergence bin. **B** Mean K2P distributions within TE superfamilies. Left panel depicts Class II families, and all Class I (retrotransposons) and unknown families are on the right panel. LTR superfamilies harbor some of the least divergent TE families, suggesting that this class may host some of the youngest TE

Within reconstructed TE families, nucleotide substitution levels (Kimura 2 parameters, K2P) between copies and their consensus sequences allowed estimation of their relative ages and identified potentially active ones (Fig. 4A). Such “TE landscapes” are extremely helpful to pinpoint potential TE amplifications (modes in the distribution) and extinctions (valleys) within the 0–30% K2P range (beyond, the increased divergence between copies affects negatively the sensitivity of the alignments, such that TE-derived sequences are no longer recognizable). The landscape analysis revealed a heterogeneous distribution of the TE copy divergence to their consensus within and between the main TE subclasses (Fig. 4A). Most identified TE copies have a K2P divergence under 10, which is often observed in insects, and strikingly distinguishes itself from TE-rich mammalian genomes (RepeatMasker.org, [40]). While *S. oryzae*’s TE density and distribution evokes the architecture of mammalian genomes, this relatively younger TE landscape suggests higher deletion rates, and possibly a higher TE turnover rate, as observed in *Drosophila* [255, 256]. LINEs and DNA transposons have the wider spectrum of divergence levels, suggesting an aggregation of distinct dynamics for the TE families present in *S. oryzae.* By contrast, the rare LTR copies identified appear to be the most homogeneous within families, with only a few substitutions between copies and their consensuses, suggesting a very recent amplification in this subclass. Finally, unknown TEs share a large part of their K2P distribution with TIR elements, though relatively less divergent from their consensus sequences as a whole. A breakdown of the K2P distributions at the superfamily level reveals specific evolutionary dynamics (Fig. 4B). Diverse superfamilies, such as Tc-Mar and hAT (TIR) or RTE (LINE), show more uniform distributions, suggesting sustained activity of some of its members throughout *S. oryzae*’s genome evolution, though this could also indicate that these subfamilies could be subdivided further. As observed at the class level, all three identified LTR superfamilies (Pao, Gypsy, and Copia) show families within the lowest K2P range.

#### TEs are transcriptionally active in somatic and germline tissues

**Fig. 5.**
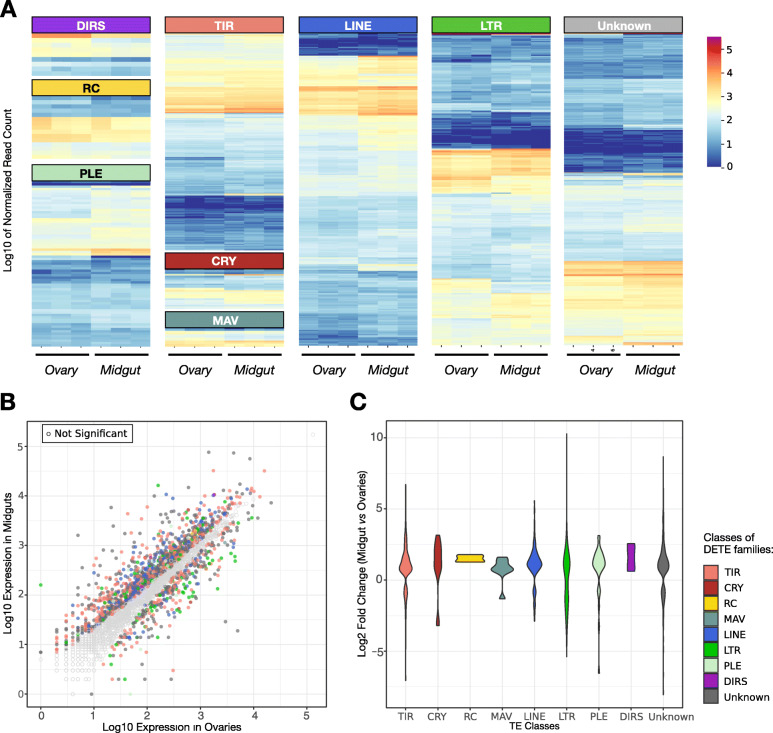
TE family expression in midguts and ovaries from *S. oryzae*. **A** Log10 normalized counts in midguts and ovaries triplicates. Normalized counts show different proportions of transcriptionally active TE families in different TE classes. **B** Log10 of base mean average expression of TE families in ovaries and midguts from three biological replicates. Depicted in color only TE families which had differential expression between ovary and gut tissues (padj< 0.05, |log2FC| > 2). Most TE families are upregulated in midguts compared to ovaries. **C** Distribution of all significant (padj< 0.05). Log2FC depicts specifically deregulated TE classes in each tissue. LTR elements are predominantly upregulated in ovaries

The TE K2P landscape suggests that LTR elements as well as some LINE families and several Class II subclasses are among the youngest, and thus potentially active. In order to estimate the transcriptional activity of *S. oryzae*’s TE families, we have produced somatic (midgut) and germline (ovary) transcriptomic data. While germline tissues allow identification of potential TE families capable of producing vertically transmitted new copies, TE derepression in somatic tissues represents the potential mutational burden due to TEs. The expression of TE families varied extensively within a class and the proportion of transcriptionally active/inactive TE families between classes was also distinct (Fig. 5A). In total, 1594 TE families were differentially expressed between ovary and midgut tissues (Fig. 5B, Additional file 5); of which 329 have an absolute log2 fold change higher than 2 (71 downregulated and 258 upregulated in midgut). In total, we detected 360 TE families downregulated in midgut when compared to ovaries: A much larger set of upregulated TE families was detected in midgut when compared to ovaries (1 236), illustrating the tighter regulation of TE copies in germline tissues. Moreover, the distribution of log2 fold changes were similar between TE subclasses but different for LTRs, which had a higher proportion of upregulated TE families in ovaries compared to other classes (Fig. 5C. Kruskall and Wallis rank-sum test: *H* = 36.18, *P* < 0.01; LTR vs. LINE, Class II or Unknown: Dunn’s test: *P*-adj < 0.01). In conclusion, the large TE compartment in *S. oryzae* shows abundantly expressed TE families, and tissue-specific expression patterns.

To estimate the TE transcriptional load imposed on *S. oryzae*, we computed the percentage of total RNAseq poly-A enriched reads mapping to TE consensus sequences in gut and ovaries (Additional file 2: Figure S4). Around 5% of the midgut transcriptome corresponds to TE sequences, while such reads represent only ~ 2% of ovarian transcriptomes, reinforcing the tighter regulation of TEs in germ tissues. We compared such transcriptional burden to a TE-poor (*D. melanogaster*, ≈12%*)* and a TE-rich (*Ae. albopictus ≈*50%*)* genome, using similar technology in equivalent tissues (adult midgut, see “Methods”). It is important to note that, despite being a TE-poor genome, *D. melanogaster* harbors many young LTR elements that have been recurrently shown to transpose [257]. We did not detect a direct correlation between genomic TE content and TE expression (Additional file 2: Figure S4). *S. oryzae* bears the highest proportion of RNAseq reads mapped against TE consensus sequences (≈5%), followed by *D. melanogaster* (≈1%) and *Ae. albopictus* (≈0.01%). Henceforth, not only is *S. oryzae* a TE-rich genome, but the transcriptional load from TEs is higher than in other TE-rich genomes (*Ae. albopictus)*, and in genomes harboring young and active TE copies (*D. melanogaster*, [38, 258]).

Finally, it is important to note that while transcriptional activation of TE copies may have an impact on the host genome, it does not indicate high transposition and therefore higher mutation rates. The high transcriptional load of *S. oryzae* compared to other species might stem from differences in TE regulation. In insects, TEs are mainly silenced by small RNAs and repressive chromatin marks [259]. More specifically, piwi-interacting RNAs (piRNAs) are able to target post-transcriptional repression of TEs, and guide chromatin silencing complexes to TE copies [259–261]. Therefore, we have annotated genes implicated in small RNA biogenesis and found that all three pathways (piRNAs but also microRNAs and small interfering RNAs biogenesis pathways) are complete (Additional file 2: Supplemental Note 9). Genes involved in piRNA biosynthesis are expressed mainly in ovaries and testes, while somatic tissues (midgut) show smaller steady-state levels (Additional file 2: Supplemental Note 9 [41, 94, 259, 260, 262–284]), suggesting the piRNA pathway is potentially functional in *S. oryzae* ovaries, and could efficiently reduce transposition.

#### TE content is variable among *Sitophilus* species

**Fig. 6.**
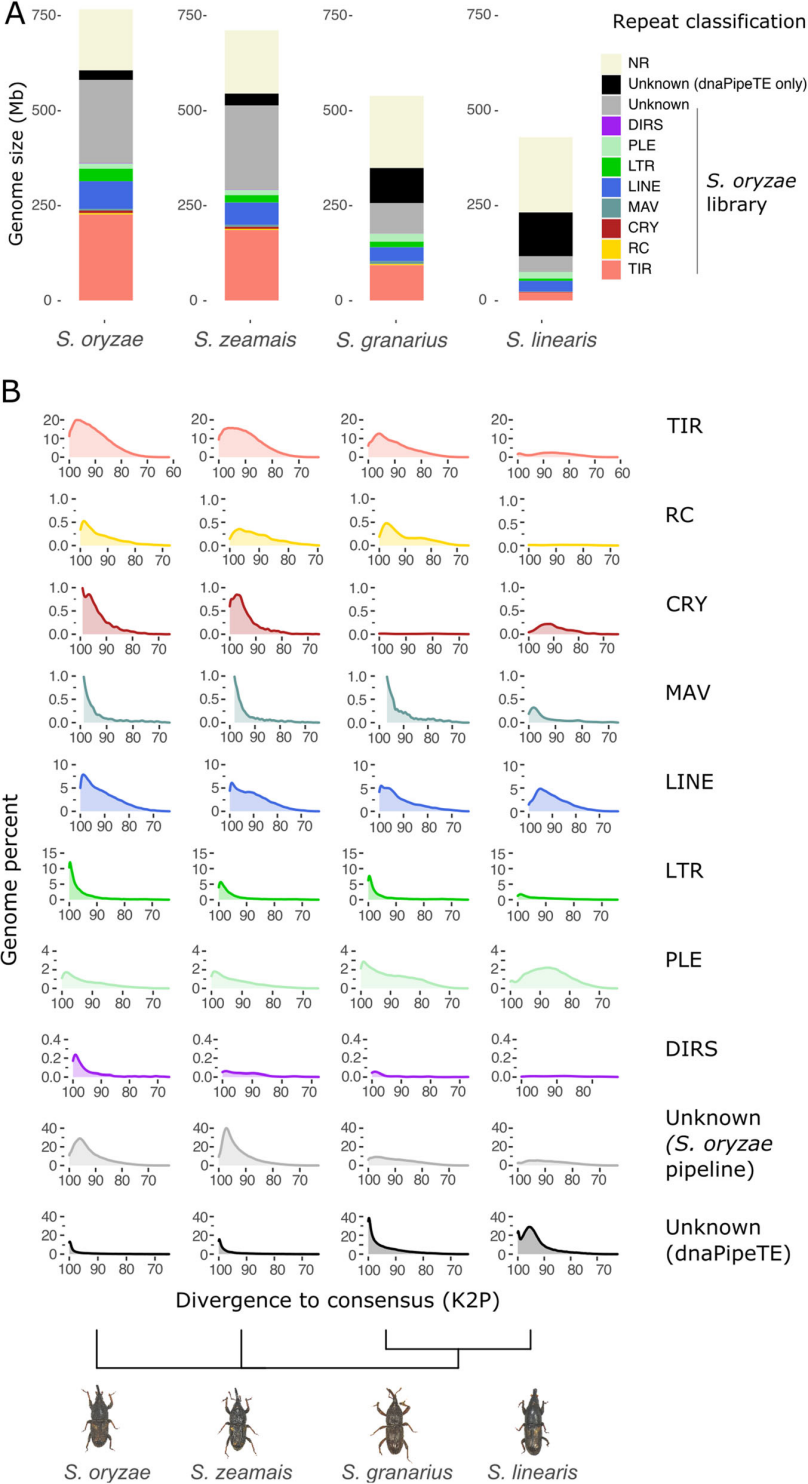
TE landscape across *Sitophilus* species. **A** Proportion of TE per species estimated from short reads with dnaPipeTE and a custom TE library including Repbase (release 2017) and annotated TE consensus discovered in *S. oryzae*. *S. oryzae*, *S. zeamais*, and *S. granarius* harbor similar TE content, while *S. granarius* presents a smaller TE load, and *S. linearis* harbors the smallest TE content and the higher proportion of unknown repeats. The proportion of unknown repeats only found by dnaPipeTE (black) increases from *S. oryzae* to *S. linearis* with the phylogenetic distance. **B** Distribution of divergence values between raw reads and repeats contig assembled with dnaPipeTE (blastn) across four *Sitophilus* species. *S. oryzae* appears to share its TE landscape with *S. zeamais* and *S. granarius*, but the three species display a distinct repeatome than *S. linearis*, in spite of their phylogenetic proximity. SO2: *S. oryzae*’s TE library produced in this analysis, DPTE: DNApipeTE TE annotation (repeats only found by dnaPipeTE)

Cereal weevils are part of the Dryophthoridae family that includes more than 500 species. Very little is known about genome dynamics in this massive phylogenetic group, and *Sitophilus* species divergence is estimated to the Neogene (2.5–25 Ma) [285] . Because of the unusual high TE copy number and landscape observed in *S. oryzae*, we analyzed three other closely related species namely *Sitophilus zeamais*, *Sitophilus granarius*, and *Sitophilus linearis*. We produced low-coverage sequencing and estimated the TE content from raw reads using our annotated *S. oryzae* TE library with dnaPipeTE [47]. Remarkably, among *Sitophilus* species, repeat content is variable (Fig. 6A), with *S. linearis* harboring the smaller repeat load (≈54%) compared to *S. oryzae* (≈80%), *S. zeamais* (≈79%), and *S. granarius* (≈65%). Most importantly, Class II (DNA) elements of *S. oryzae* are nearly absent from *S. linearis*, and no recent burst of LTR elements is observed, contrary to the other *Sitophilus* species, suggesting alternative TE evolutionary histories (Fig. 6B). It is important to note that our analysis is biased toward *S. oryzae*, as the library used to annotate the TEs in the other *Sitophilus* species stems from automatic and manual annotation of the *S. oryzae* genome.

Overall, the comparison of TE content in closely related species highlights the influence of phylogenetic inertia, but reveals a possible TE turnover in the *S. linearis* lineage. In addition to the regulation mechanisms that strongly contribute to TE amount and variation, TE accumulation is conditioned by the drift/selection balance in populations. Indeed, effective population size has been suggested to be a major variable influencing TE content, as small, inbred, or expanding populations suffer drift, allowing detrimental insertions to stay in the gene pool and thus favor TE fixation [286]. Such hypotheses should be addressed in the future, especially on recently sequenced TE-rich but rather small (< 1 Gbp) genomes such as *S. oryzae.*

#### Endosymbionts might impact TE transcriptional regulation

**Fig. 7.**
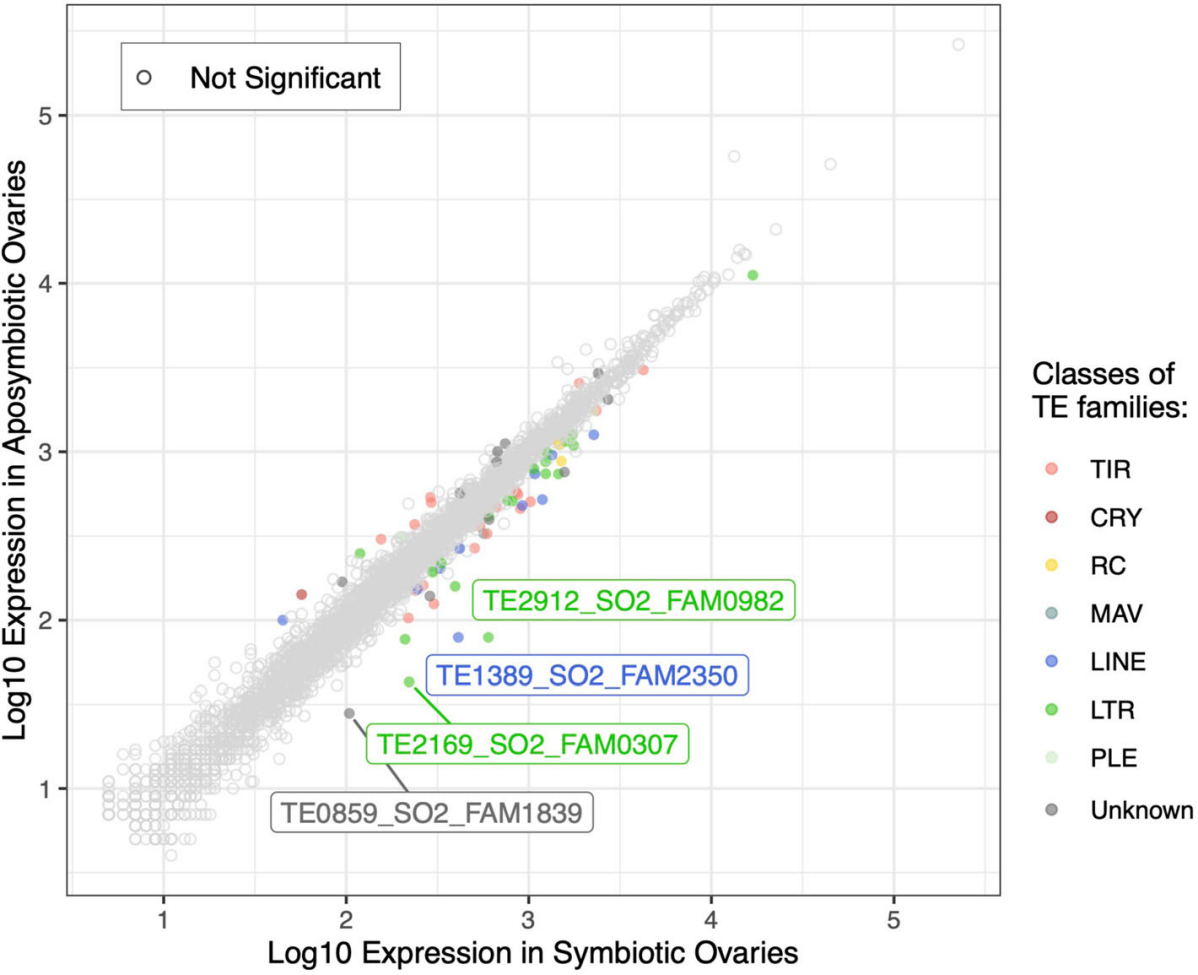
Differentially expressed TE families between symbiotic and aposymbiotic *S. oryzae* ovaries. Log10 of base mean average expression of TE families in symbiotic vs aposymbiotic ovaries from two biological replicates. Depicted in color only TE families which had differential expression between both ovary types (padj< 0.05, |log2FC| > 2). Two LTR elements and one LINE element are upregulated (log2FC > 2) in symbiotic ovaries

The four *Sitophilus* species studied have different ecologies. *S. oryzae* and *S. zeamais* infest field cereals and silos, while *S. granarius* is mainly observed in cereal-containing silos. *S. linearis*, however, lives in a richer environment, i.e., tamarind seeds. In association with their diets, the interaction of *Sitophilus* species with endosymbiotic bacteria differs: the cereal weevils (*S. oryzae*, *S. zeamais*, and *S. granarius*) harbor the intracellular gram-negative bacteria *S. pierantonius*, albeit at very different loads. While *S. oryzae* and *S. zeamais* show high bacterial load, *S. granarius* has a smaller bacterial population [111]. In contrast, *S. linearis* has no nutritional endosymbionts, in correlation with its richer diet. We wondered whether the presence of intracellular bacteria impacts TE regulation, and took advantage of artificially obtained aposymbiotic *S. oryzae* animals to search for TE families differentially expressed in symbiotic versus aposymbiotic ovaries. There were 50 TE families upregulated in symbiotic ovaries compared to artificially obtained aposymbiotic ones, while 15 families were downregulated (Fig. 7 and Additional file 5). Only three families presented an absolute log2 fold change higher than 2: one LINE and two LTR/Gypsy elements. The three of them were upregulated both in symbiotic *versus* aposymbiotic ovaries, and in ovaries *versus* midgut (Additional file 5), suggesting that such elements have tissue specificity, and their expression is modulated by the presence of intracellular bacteria. Such TE families would be ideal candidates to further study the crosstalk between host genes, intracellular bacteria and TE transcriptional regulation. It is important to note that the process used to obtain aposymbiotic insects relies in heat treatments that could impact overall transcriptional regulation, and henceforth, the TEs differentially expressed between symbiotic and aposymbiotic ovaries could stem from such treatment and not from the lack of endosymbionts. In order to confirm the link between intracellular bacteria and TE regulation, it is mandatory to deplete insects of their endosymbionts using other methods, as antibiotic treatment, and reassess TE expression in aposymbiotic individuals.

## Conclusion

The success of obtaining a TE-rich genome assembly complete enough to understand genome architecture and regulatory networks relies on the use of multiple sequencing platforms [287]. Here, we describe the first assembly of the repeat-rich (74%) *S. oryzae* genome, based on a combination of long- and short-read sequencing, and a new assembly method, WENGAN [288]. While this first assembly reaches quality standards similar to other coleopteran species (Table 1), it is important to stress that new sequencing methods have emerged in order to improve genome assemblies, including linked reads and optical mapping [287].

We uncovered around 74% of repeated sequences in the *S. oryzae* genome, mostly TE families. While the TE landscape is marked by a wealth of Class II elements, especially non-autonomous MITE elements, ~ 21% of the genome is composed of unknown repeats. Large duplicated gene families can be present in such a category, but it is tempting to speculate that the majority is composed of novel Class II elements. Indeed, unknown and TIR elements share the same K2P landscapes, and many Class II elements have only been detected through an inverted repeat search for TIRs, and not proteins, excluding therefore TE copies old enough that TIRs are too divergent to be recognized. Moreover, we have shown that many TE families in *S. oryzae* are present in the transcriptome, suggesting that several families can be transcriptionally active. How such TE families are able to escape host silencing remains unknown. It seems obvious today that insect models such as *D. melanogaster* only represent a small window on the complex biology and evolution of TEs, and the sequencing and annotation of species with high TE content—while challenging [289]—is key to understanding how genomes, their size, their structure, and their function evolve. In conclusion, *S. oryzae* constitutes an excellent model to understand TE dynamics and regulation and the impact on genome function.

*Sitophilus* species not only differ in their TE landscape, but also in their ecology and as a consequence, their association with intracellular bacteria. Comparison of TE content within the *Sitophilus* genus shows variable TE amount and diversity. In addition, intracellular bacterium impacts transcription of specific TE families in ovaries. The molecular mechanisms behind the co-evolution between an insect, its endosymbiotic bacterium, and TEs remain unexplored. The impact of intracellular bacteria on host genomes is poorly studied, and the *Sitophilus* genus offers a simpler experimental setting, with a single intracellular bacterium present within specific host cells [19, 112], and a well-established knowledge of host-bacteria interaction [111, 147, 148, 193, 290].

## Methods

### DNA extraction and high-throughput sequencing

Individuals of both sexes of *S. oryzae* were reared on wheat grains at 27.5 °C with 70% relative humidity. The aposymbiotic strain was obtained by treating the symbiotic strain during one month at 35 °C and 90% relative humidity as previously described [291]. This strain is viable, is fertile, and was raised in the same conditions as the symbiotic strain. The aposymbiotic status was confirmed by PCR and histology. Male and female adults of *S. oryzae* were used for DNA extraction. Only the gonads were used to minimize DNA contamination from its diet, which could be still present in the gut. The reproductive organs were obtained from aposymbiotic adults and a DNA extraction protocol specific for *Sitophilus* weevils was performed. DNA extractions were performed using a STE buffer (100 mM NaCl, 1 mM Na_2_EDTA pH 8, 10 mM Tris HCl pH 8). Tissues were homogenized in STE buffer, then treated successively by SDS 10%, proteinase K, and RNase. Briefly, genomic DNA was purified by two successive extractions with phenol:chloroform:isoamyl alcohol (25/24/1) followed by extraction with 1 vol of chloroform:isoamyl alcohol (24/1). Genomic DNA was then precipitated by 0.7 vol isopropanol. After washing the pellet with 70% ethanol, genomic DNA was recovered in TE (1 mM EDTA, 10 mM Tris HCl pH 8) buffer. Using this protocol, we obtained six different DNA samples: four from males and two from females. Each sample corresponds to the genomic DNA from 20 individuals. Five additional DNA samples were obtained using a high molecular weight DNA extraction protocol consisting of a single phenol:chloroform:isoamyl alcohol (25/24/1) extraction step from the genomic DNA of 100 males. The DNA concentration in each of these samples was quantified using a NanoDrop spectrophotometer (Thermo Fisher Scientific, Waltham, MA, USA).

Sequencing was performed using a combination of Illumina, PacBio, and Nanopore technologies (Additional file 1). For each sex, two Illumina libraries were generated: one paired-end library with an average fragment size of 500 bp and one mate pair library with an average fragment size of 5 kbp. The libraries were sequenced using an Illumina HiSeq 2000 platform with the V3 chemistry and a read size of 101 bp; the paired-end (PE) libraries were sequenced at the “Génomique & Microgénomique” service from ProfileXpert (Lyon, France) while the mate paired (MP) were sequenced at Macrogen (Seoul, South Korea). Two male samples were used to build (i) an Illumina library with an average fragment size of 200 bp which was sequenced on a HiSeq 2500 instrument using the V4 chemistry and a read size of 125 bp, and (ii) a PacBio library sequenced on seven SMRT cells using the P6-C4 chemistry. These two libraries were sequenced at KeyGene (Wageningen, The Netherlands). Finally, five male samples were used to build Nanopore libraries with the SQK-LSK109 kit and without DNA fragmentation step. The libraries were independently sequenced on five MinION R9.4 flow cells. These libraries were built and sequenced at the sequencing platform of the IGFL (Institut de Génomique Fonctionnelle de Lyon, Ecole Normale Supérieure de Lyon, France). Statistics and accession numbers from all the sequencing runs are listed in the Additional file 1.

### Genome assembly and annotation

First, the Illumina reads were error-corrected using BFC release 181 [292]. The PacBio and Nanopore reads were error-corrected using LORDEC v0.9 [293] with the error-corrected Illumina overlapping PE reads, a k-mer size of 19 and solidity threshold of 3. Overlapping reads were then merged using FLASH2 v2.2 [294]. Based on the merged Illumina reads, a first short-read assembly was produced using a modified version of MINIA v3.2.1 [295] with a k-mer length of 211. A hybrid assembly was then performed using WENGAN v0.1 [288] on the MINIA short-read assembly and the raw Nanopore reads. The resulting assembly was polished using two rounds of PILON v1.23 [296] using the error-corrected Illumina overlapping PE reads and the --diploid option. A first scaffolding was then performed with two rounds of FAST-SG v06/2019 [297] and SCAFFMATCH v0.9 [298] with the error-corrected Illumina MP, Illumina PE, PacBio, and Nanopore libraries. The LR_GAPCLOSER algorithm v06/2019 [299] was used for the gap-filling step using the error-corrected PacBio and Nanopore libraries. An additional scaffolding step was performed using RASCAF v1.0.2 [300] with the available RNAseq libraries from the Sequence Read Archive (SRX1034967-SRX1034972 and SRX3721133-SRX3721138). The resulting scaffolds were then gap-filled using a new round of LR_GAPCLOSER as previously described followed by two rounds of SEALER v2.1.5 [301] using the error-corrected Illumina overlapping PE reads and k-mer sizes of 64 and 96. Two rounds of PILON, as previously described, were performed to produce the final assembly. During the assembly process, we assessed haplotig contamination by using purge_haplotigs [302] and purge_dups [303]. No diploid peak nor significant haplotig contamination was observed. Quality of the assembly was assessed by computing several metrics using (i) QUAST v5.0.2 [304] with a minimal contig size of 100 bp and the --large and -k options, (ii) BUSCO v4.0.5 [50] using the Insecta ODB10 database and the -geno option, and (iii) KMC v3.0.0 [305] to evaluate the percentage of shared 100-mers between the assembly and the merged Illumina reads. Genome size prediction was performed with GenomeScope v2.0 [58], findGSE v1.94.R [60], and gce v1.0.2 [59] based on 21-mer histograms generated by JellyFish v2.2.10 [306] on the R1 reads from error-corrected Illumina overlapping PE library.

Three contaminant scaffolds corresponding to the mitochondrial genome and an artifact were removed from the assembly prior to the annotation step. The “NCBI *Sitophilus oryzae* Annotation Release 100” was produced using the NCBI Eukaryotic Genome Annotation Pipeline v8.2.

### Low-coverage genome sequencing of other *Sitophilus* species

Twenty pairs of ovaries were dissected from *S. oryzae*, *S. zeamais*, *S. granarius*, and *S. linearis* females. Ovaries were homogenized in 100 mM NaCl, 1 mM EDTA pH 8, and 10 mM Tris-HCl pH 8 using a small piston. Proteinase K digestion followed in the presence of SDS for 2 h at 55 °C with shaking and for 1 h at 37 °C with RNAse A. A typical phenol chloroform extraction was then performed and genomic DNA was isopropanol precipitated. Eight whole genome sequencing libraries with a median insert size of 550 bp were constructed using the Illumina TruSeq DNA PCR-free sample preparation kit (Illumina, San Diego, CA, USA), according to the manufacturer’s protocols. Briefly, 2 μg of each gDNA was sheared using a Covaris M220 Focused-ultrasonicator (Covaris, Inc. Woburn, MA, USA), end-repaired, A-tailed, and adapter ligated. Library quality control was performed using the 2100 Bioanalyzer System with the Agilent High Sensitivity DNA Kit (Agilent Technologies, Santa Clara, CA, USA). The libraries were individually quantified via qPCR using a KAPA Library Quantification Kits (Kapa Biosystems, Wilmington, MA, USA) for Illumina platforms, then they were pooled together in equimolar quantities and sequenced in a MiSeq sequencing system. 2 × 300 paired-end reads were obtained using a MiSeq Reagent Kits (600-cycles).

### TE library construction

In order to annotate the *S. oryzae* repeatome, we collected and combined cutting-edge bioinformatic tools to (i) create and (ii) classify a non-redundant library of repeated elements (Additional file 2: Figure S1). First, we separately ran RepeatModeler2 (v2.0.1) [244] and EDTA v1.7.8 [245] on the assembled genome. Together, these programs include most of the recent and long-trusted tools used to detect generic repeats, but also include specific modules, such as for LTR and TIR elements. Preliminary analyses of the *S. oryzae* genome with RepeatModeler1 [307] and dnaPipeTE (v1.3) [47] suggested a rather large fraction of Class II DNA elements with terminal inverted repeats (TIRs). Thus, MITE-Tracker [308] was incorporated in our pipeline and ran independently on the genome assembly using 1- and 2-kbp size cutoffs to detect Class II elements harboring TIRs with high sensitivity. Following this initial step, 15,510 consensus sequences obtained from RM2, EDTA, and the two runs of MITE-tracker were successively clustered using MAFFT (v6.864b) [65], Mothur (v.1.45.2) [309], and Refiner [307] to reduce redundancy in the repeat library to a total of 2754 consensus sequences (Additional file 2: Figure S1A, https://github.com/clemgoub/So2). Then, we inspected the quality of the raw library by calculating the genomic coverage of each consensus. We ran the library against the genome using RepeatMasker (v4.1.1) (52) and implemented a simple algorithm “TE-trimmer.sh” to trim or split a consensus sequence wherever the genomic support drops below 5% of the average consensus coverage (Additional file 2: Figure S1A, https://github.com/clemgoub/So2). To mitigate any redundancy generated by the splitting, the newly trimmed library was clustered before being re-quantified using RepeatMasker (v4.1.1) [307]. At this step, we removed any consensus under 200 bp and represented by less than the equivalent of two full-length copies (in total bp). In addition, TAREAN (RepeatExplorer2 v0.3.6) [310] was used to detect and quantify candidate satellite repeats. We obtained an ab initio repeat library of 3950 consensus sequences automatically generated (Additional file 2: Figure S1A).

To refine and improve the quality of the TE consensus sequences, we then turned it over to DFAM [311] who processed the ab initio library following their recent guidelines [312]. First, any sequences mostly composed of tandem repeats were removed using a custom script to remove any sequences that were greater than 80% masked and/or had a sequence less than 100 bp. To generate seed alignments for each consensus, the consensus sequences were used as a search library for RepeatMasker (v4.1.1) to collect interspersed repeats. Seed alignments in the form of Stockholm files were generated using the RepeatMasker output. To extend potentially truncated elements, the instances in the Stockholm file for each model were extended into neighboring flanking sequences until the alignment was below a threshold equivalent to ~ 3 sequences in agreement. More specifically, all sequences are extended using full dynamic programming matrices using an improved affine gap penalty (default: − 28 open, − 6 extension) and a full substitution matrix (default: 20% divergence, 43% GC background). The termination of extension occurs when the improvement by adding a further column to the multiple alignment does not exceed 27 (with default scoring system). This is equivalent to a net gain of ~ 3 sequences in agreement. Following extension, the new consensus were collected and consensus sequences greater than 80% similar for 80% of their length were considered duplicates and only one consensus was kept.

Upon completion, we used RepeatMasker to quantify the improved library. We selected the top 50 elements (by abundance in the genome) represented in each of the “LTR,” “LINE,” “Class II,” and “Unknown” classes for manual inspection (these categories represent the 4 most abundant classes of repeats in the *S. oryzae* genome). While most consensus sequences were correctly extended and annotated (200), we noticed some cases of over-extension with LTR (consensus doubled in size) and flagged others with non-supported fragments for further trimming (Additional file 4 | tab 1). Once our quality check completed and the sequences curated, we removed fragments with 100% identity against a previously established consensus (Additional file 4 | tab 2). The final TE library contains 3399 sequences to classify.

The classification of the final repeat library was done in successive rounds combining homology and structure methods (Additional file 2: Figure S1B). Before the final TE library was completed, we manually curated and annotated the sequences of 31 transposable elements and satellites among the most represented in *S. oryzae*. These 31 high-confidence consensus sequences are added to the libraries used by the annotation programs described below and Repbase v.2017 [313]. We searched for nucleotide homology using RepeatMasker (v4.1.1 [307]) with -s “-slow” search settings. Best hits were chosen based on the highest score at the superfamily level allowing non-overlapping hits of related families to contribute to the same hit. In addition, we used blastx [94] to query each consensus against a curated collection of TE proteins (available with RepeatMasker), as well as those identified in the 31 manual consensus sequences. We kept the best protein hit based on the blastx score. Based on the 200 consensus sequences manually inspected (see above), we set a hit length / consensus size threshold of 0.08 (RepeatMasker) and 0.03 (blastx) to keep a hit. In our hands, these thresholds were conservative to automate the classification. As an alternate homology-based method, we also ran RepeatClassifier (RepeatModeler2, v2.0.1). Finally, because DNA elements are often represented by non-autonomous copies (unidentifiable or absent transposase), we further used einverted to flag terminal inverted repeats located less than 100 bp of the ends of each sequence. The complete library of 3399 consensus sequences was first annotated at the subclass level (see DFAM taxonomy: https://dfam.org/classification/tree) if two out of RepeatMasker, RepeatClassifier, and blastx annotations agreed. Further, the same rule was applied for the superfamilies if possible. At this stage, consensus sequences without annotation by homology but with TIRs as flagged by einverted were classified as TIR and all other sequences classified as Unknown. We further divided the subclass “DNA” into “MAV” (Mavericks), “RC” (Rolling circle/Helitron), “CRY” (Crypton), and “TIR” (terminal inverted repeats). Finally, the classifications automatically given as “Unknown” to 16/274 manually inspected consensus sequences were replaced to match the manually reported classification.

In order to remove potential multi-copy gene families which would have made their way to the TE library, we searched for non-TE conserved protein domains using NCBI’s CDD search with all peptides ≥ 100 AA extracted from the unknown repeats [314]. Significant hits (*P* ≤ 0.01) against known TEs and viruses were removed and all other left consensus were removed from the TE library. In conclusion, there are 21% unknown repeats, and the number of total TE consensus sequences in *S. oryzae* is 3361. The data can be obtained from 10.5281/zenodo.5128603.

In order to assess the relevance of our custom TE analysis pipeline, we ran and compared the unfiltered outputs (out.tbl file) of RepeatMasker v4.1.1 using either the TE library produced by RepeatModeler 2.0.1, EDTA v1.7.8, or our final library. An optimized TE library should minimize the total number of consensus while being able to capture as much TE in the genome as possible. Thus, we compared the total number of consensus built in each library as well as the total percent of the genome masked by each respective library.

### Estimation of the repeat content

The total repeat content of the *S. oryzae* genome was analyzed using RepeatMasker (v.4.1.1) and our classified library of 3361 consensus sequences and the following parameters: -s -gccalc -no_is -cutoff 200. The subsequent alignments were parsed with the script “parseRM.pl” [315] https://github.com/4ureliek/Parsing-RepeatMasker-Outputs) to remove hits overlap and statistically analyzed with R version 4.0.2.

### Genomic distribution of TE copies

The distribution of TE copies across the *S. oryzae* genome was assessed using two different approaches over six different genomic regions namely TSS ± 3 kbp, 5′ UTRs, exons, introns, 3′ UTRs, and intergenic regions. Briefly, the coverage of all TE copies was computed over a sliding window of 100 bp across the whole genome sequence using the makewindows and coverage tools from the bedtools package [280] and the bedGraphToBigWig UCSC gtfToGenePred tool. Then the different genomic regions were retrieved from the *S. oryzae* annotation file (GFF format) using the gencode_regions script (https://github.com/saketkc/gencode_regions) and the UCSC gtfToGenePred tool (https://github.com/ENCODE-DCC/kentUtils). A matrix containing the TE coverage per genomic region was generated using the computeMatrix tool from deepTools [279] and used to generate metaplots using the plotProfile tool.

### TE landscapes

The relative age of the different TE families identified in the genome assembly was drawn performing a “TE landscape” analysis on the RepeatMasker outputs. Briefly, the different copies of one TE family identified by RepeatMasker are compared to their consensus sequence, and the divergence (Kimura substitution level, CpG adjusted, see RepeatMasker webpage: http://repeatmasker.org/webrepeatmaskerhelp.html) is calculated. The TE landscape consists of the distribution of these divergence levels. In the end, the relative age of a TE family can be seen as its distribution within the landscape graph: “older” TE families tend to have wider and flatter distribution spreading to the right (higher substitution levels) than the “recent” TE families, which are found on the left of the graph and have a narrower distribution. TE landscapes were drawn from the RepeatMasker output parsed with the options -l of “parseRM.pl.” We report here the TE landscape at the level of the TE subclass (LINE, LTR, TIR, CRY, MAV, DIRS, PLE, RC, and Unknown).

### dnaPipeTE comparative analysis in *Sitophilus* species

To compare the TE content of *S. oryzae* to four related species of *Sitophilus* (*S. granarius*, *S. zeamais*, *S. linearis*), we used dnaPipeTE v.1.3 [47]. dnaPipeTE allows unbiased estimation and comparison of the total repeat content across different species by assembling and quantifying TE from unassembled reads instead of a linear genome assembly. Reads for *Sitophilus* species were produced as described above. Using our new classified library (3 390 consensus) as TE database in dnaPipeTE, we were further able to identify the phylogenetic depth of the repeat identified in *S. oryzae*.

### RNA sequencing and TE expression analysis

Individuals of both sexes of *S. oryzae* were reared on wheat grains at 27.5 °C with 70% relative humidity. Ten midguts and ovaries from 10-day-old adults were dissected in diethylpyrocarbonate-treated Buffer A (25 mM KCl, 10 mM MgCl_2_, 250 mM Sucrose, 35 mM Tris/HCl, pH 7.5). RNA was extracted in triplicates with RNAqueous-Micro (Qiagen), following the manufacturer recommendations. Single-indexed libraries were built using the SENSE mRNA-Seq Library Prep Kit V2 (Lexogen), following the manufacturer recommendations. Libraries were then pooled in an equimolar range and sequenced using high-throughput reagents on an Illumina NextSeq 500 (86 bp in single end). Raw sequencing reads were deposited at SRA archive BioProject PRJNA746240. Adapter sequences and low-quality reads were filtered out with Trimmomatic (v0.36) [316] and clean reads were aligned to the *S. oryzae* genome with STAR aligner (v2.5.4b, [317]) and featureCounts from subread package [318] to obtain gene counts. We also used the STAR aligner in single-end mode to map the clean reads against all TE copies extracted from the genome with the following options: --outFilterMultimapNmax 100 --winAnchorMultimapNmax 100 --outMultimapperOrder Random --outSAMmultNmax 1. The mapped bam files were used as input to TEtools software [319] to determine TE family expression. Genes and TE family counts were used as input for DESEq2 package (v1.30) [320] to determine differential TE expression between ovary vs gut tissues as well as ovaries from symbiotic and aposymbiotic weevils. Differentially expressed TEs were defined whenever the adjusted *p* value was smaller than 0.05 and log2 fold change was higher than 1 or smaller than − 1. We used the aforementioned STAR alignment parameters to map transcriptomic sequencing reads from midgut and ovaries of *S. oryzae* (Accession: SRX1034971, SRX1034972, and reads from this study deposited under the BioProject ID PRJNA746240), *D. melanogaster* (Accession: SRX029389, and SRX045361), and *Ae. albopictus* (Accession: SRX1512976, SRX1898481, SRX1898483, SRX1898487, SRX3939061, and SRX3939054) against the TE consensus sequences for each species.

## Supplementary Information

**Additional file 1: **Summary of sequencing libraries produced for *S. oryzae.*

**Additional file 2:.** Supplementary notes, supplementary figures, and small tables.

**Additional file 3:.** Large supporting tables and datasets.

**Additional file 4:.** Transposable elements annotation tables.

**Additional file 5:.** STAR and TEtools mapping statistics.

## Data Availability

Data generated and analyzed during this study are included in the published article, its additional files and publicly available repositories. This Whole Genome Shotgun project has been deposited at DDBJ/ENA/GenBank under the accession PPTJ00000000 [321]. The version described in this paper is version PPTJ02000000. The assembly can be visualized, along with gene models and supporting data, on a dedicated genome browser (https://bipaa.genouest.org/sp/sitophilus_oryzae/). Raw reads from low-coverage genome sequencing of *S. zeamais*, *S. granarius*, and *S. linearis* have been deposited at NCBI Sequence Read Archive (SRA) under the BioProject accessions PRJNA647530 [322], PRJNA647520 [323], and PRJNA647347 [324] respectively. TE annotation (GFF) and consensus sequences can be found at 10.5281/zenodo.4570415 [325]. RNAseq reads obtained in this manuscript were deposited under the BioProject accession PRJNA746240 [326]. Bisulfite-seq reads have been deposited at NCBI SRA, under the BioProject accession PRJNA681724 [327].

## Abbreviations

AMPs: Antimicrobial peptides
CPs: Cuticle proteins
HGT: Horizontal gene transfer
IMP: Inosine monophosphate
K2P: Kimura 2 parameters
LINE: Long-interspersed element
LTR: Long terminal repeat
MAMPs: Microbial associated molecular patterns
MITEs: Miniature inverted repeat elements
ORs: Odorant receptors
PRR: Pattern recognition receptors
PLE: Penelope-like
RC: Rolling circle
SINE: Short interspersed element
TIR: Terminal inverted repeat
TSS: Transcription start sites
TEs: Transposable elements
TTSS: Type three secretion systems
UTR: Untranslated regions
UMP: Uridine monophosphate
